# Modifiable dementia risk score to study heterogeneity in treatment effect of a dementia prevention trial: a post hoc analysis in the preDIVA trial using the LIBRA index

**DOI:** 10.1186/s13195-018-0389-4

**Published:** 2018-06-30

**Authors:** Tessa van Middelaar, Marieke P. Hoevenaar-Blom, Willem A. van Gool, Eric P. Moll van Charante, Jan-Willem van Dalen, Kay Deckers, Sebastian Köhler, Edo Richard

**Affiliations:** 1 Department of Neurology, Academic Medical Center (AMC), Meibergdreef 9, 1105 AZ Amsterdam, the Netherlands; 2 Department of Neurology, Donders Institute for Brain, Cognition and Behaviour, Radboud University Medical Center, Nijmegen, the Netherlands; 3 Department of General Practice, Amsterdam Public Health Research Institute, Academic Medical Center (AMC), Amsterdam, the Netherlands; 4 Department of Psychiatry and Neuropsychology, Alzheimer Center Limburg, Maastricht University, Maastricht, the Netherlands

**Keywords:** Dementia, Prevention, Prognosis, Risk factors, Randomised controlled trial, Patient selection

## Abstract

**Background:**

Selecting high-risk participants for dementia prevention trials based on a modifiable dementia risk score may be advantageous, as it increases the opportunity for intervention. We studied whether a multi-domain intervention can prevent all-cause dementia and cognitive decline in older people across three different levels of a modifiable dementia risk score.

**Methods:**

Prevention of Dementia by Intensive Vascular Care (preDIVA) is a randomised controlled trial studying the effect of multi-domain vascular care during 6–8 years on incident all-cause dementia in community-dwelling people aged 70–78 years. For this post hoc analysis, we stratified preDIVA participants in tertiles based on their baseline LIfestyle for BRAin Health (LIBRA) index, a modifiable dementia risk score. With Cox proportional hazards regression, the intervention effect on dementia was assessed. The effect on cognition was measured every 2 years with the Mini-Mental State Examination and Visual Association Test.

**Results:**

Dementia developed in 220 of 3274 (6.7%) participants. In participants with a low, intermediate and high LIBRA index, the hazard ratio (HR) of the intervention on incident dementia was respectively 0.71 (95% CI 0.45–1.12), 1.06 (95% CI 0.66–1.69) and 1.02 (95% CI 0.64–1.62). Also, when adding the non-modifiable risk factors age, education and sex to the index, results were comparable (respectively HR 0.88, 95% CI 0.54–1.43; HR 0.91, 95% CI 0.57–1.47; HR 0.92, 95% CI 0.59–1.41). There was no statistically significant intervention effect on cognition during follow-up across the LIBRA groups.

**Conclusions:**

In the preDIVA study population aged 70–78 years, the LIBRA modifiable dementia risk score did not identify a (high-)risk group in whom the multi-domain intervention was effective in preventing dementia or cognitive decline.

**Trial registration:**

International Standard Randomised Controlled Trial Number registry, ISRCTN29711771. Registered on 14 February 2006.

**Electronic supplementary material:**

The online version of this article (10.1186/s13195-018-0389-4) contains supplementary material, which is available to authorized users.

## Background

The number of dementia cases worldwide is anticipated to double over the coming two decades [1, 2]. Up to a third of Alzheimer’s disease cases may be attributable to potentially modifiable risk factors, including several vascular risk factors such as diabetes mellitus, midlife hypertension and physical inactivity [3]. This offers a window of opportunity for prevention strategies. However, selection of the optimal target population when designing a randomised controlled trial (RCT) to prevent dementia remains a challenge [4]. Results from recent RCTs suggest that interventions may be most effective in those individuals at increased risk of dementia based on the presence of one or more dementia risk factors [5–7]. In such an at-risk population the potential to improve modifiable risk factors such as hypertension and physical inactivity, and thereby prevent dementia, is higher. In addition, the higher dementia incidence rates in high-risk populations increase the study power, decreasing the total number of participants required to demonstrate a treatment effect.

A dementia risk score could be a useful tool to recruit a high-risk population for prevention trials. Most risk scores that have been developed are, however, heavily dependent on non-modifiable risk factors such as age, sex and education [8]. The LIfestyle for BRAin Health (LIBRA) index is the first, and so far only, validated dementia risk score predominantly supported by modifiable health and lifestyle factors [9]. It consists of the following 12 risk and protective factors: depression, hypertension, obesity, smoking, hypercholesterolemia, diabetes, renal dysfunction, physical inactivity, coronary heart disease, low/moderate alcohol use, cognitive activity and adherence to the Mediterranean diet. As the index reflects an individual’s potential for dementia prevention, it may identify those most responsive to an intervention.

Prevention of Dementia by Intensive Vascular Care (preDIVA) is a cluster RCT evaluating the effect of 6–8 years of nurse-led intensive vascular care on incident dementia in community-dwelling older people aged 70–78 years [5]. Overall, no preventive effect of the intervention was found. The intervention seemed more beneficial in a subgroup of individuals with untreated hypertension who adhered to the intervention. As the preDIVA intervention targets several vascular risk factors, our hypothesis was that a risk score capturing several modifiable risk factors may function even better at selecting those individuals responsive to the intervention.

Hence, our aim was to study whether a multi-domain intervention can prevent all-cause dementia and cognitive decline in older people across three different levels of a modifiable dementia risk score.

## Methods

The current study is a post hoc analysis in the preDIVA trial, which was published previously [5]. In short, the intervention comprised 4-monthly visits to a practice nurse who gave individually tailored lifestyle advice on smoking, diet, physical activity, weight and blood pressure (BP). If indicated, pharmacological treatment was started or optimised according to the prevailing guidelines on cardiovascular risk management [10]. The control condition was standard care. All community-dwelling older people aged 70–78 years registered at participating Dutch general practices were invited to participate. The only exclusion criteria were a diagnosis of dementia and/or any condition likely to hinder long-term follow-up (such as terminal illness or alcoholism). The trial is registered at the International Standard Randomised Controlled Trial Number registry (ISRCTN29711771).

### LIBRA index

The LIBRA index has been designed based on a systematic review and Delphi consensus, and has been validated in several cohorts, including a cohort aged 70–79 years [9, 11, 12]. In preDIVA, 10 out of 12 LIBRA factors were measured at baseline (Table 1). Similar to one of the previously mentioned validation studies [11], there was no information on cognitive activity or adherence to the Mediterranean diet. Data on medical history, medication use, history of smoking and alcohol use were self-reported and cross-referenced with the electronic medical record of the general practitioner. BP, weight and height (to calculate the body mass index (BMI)) were measured using standard protocols. A blood sample was drawn to measure cholesterol and creatinine levels. The 15-item Geriatric Depression Scale was used to measure depressive symptoms and the LASA Physical Activity Questionnaire to measure physical activity [13, 14]. The measures corresponding to the 10 LIBRA items (Table 1) were aligned to the previously published validation studies [9, 11]. Each item was assigned the appropriate score (Table 1) and the sum of these items formed the LIBRA index (with a maximum potential range of − 1.0 to + 12.7). Only participants with all 10 items available to calculate the LIBRA index were included in the analysis. For a secondary analysis, the LIBRA index was extended with the non-modifiable risk factors age, sex and education (Table 1), in order to make it more comparable to other available dementia risk indices [15]. This was also done in the previously published studies on the LIBRA index [9, 11].

**Table 1:** Definition of risk/protective factors in the LIBRA index and corresponding scores [11]

	Definition	Score
Modifiable risk factors		
Depression	Score ≥ 5 on the 15-item Geriatric Depression Scale	+ 2.1
Hypertension	SBP ≥ 140 mmHg, DBP ≥ 90 mmHg and/or use of antihypertensive medication	+ 1.6
Obesity	BMI ≥ 30	+ 1.6
Smoking	Current smoker	+ 1.5
Hypercholesterolemia	Total cholesterol ≥ 6.2 mmol/L or use of cholesterol-lowering medication	+ 1.4
Diabetes	Diabetes mellitus^a^	+ 1.3
Renal dysfunction	Estimated glomerular filtration rate < 60 ml/min/1.73 m^2 b^	+ 1.1
Physical inactivity	Not fulfilling World Health Organisation criteria for physical activity as measured with LASA Physical Activity Questionnaire^c^	+ 1.1
Coronary heart disease	Cardiovascular disease (defined as myocardial infarction, angina or peripheral arterial disease)^a^	+ 1.0
Low/moderate alcohol use	Alcohol use 1–14 units per week for males and 1–7 for females [21]	−1.0
Non-modifiable risk factors		
Age	Males: 70–74 years	+ 5.2
	Male: 75–78 years	+ 6.8
	Females: 70–74 years	+ 6.2
	Female: 75–78 years	+ 9.2
Education	High: ≥ 13 years	0
	Medium: 7–13 years	+ 1.4
	Low: ≤ 7 years	+ 2.7

*LIBRA* LIfestyle for BRAin Health, *SBP* systolic blood pressure, *DBP* diastolic blood pressure, *BMI* body mass index

^a^Data self-reported and cross-checked with electronic health records

^b^Estimated glomerular filtration rate calculated with the creatinine-based Chronic Kidney Disease–Epidemiology Collaboration equation [27]

^c^World Health Organisation criteria for physical activity defined as ≥ 150 min/week moderate intensity or ≥ 75 min/week vigorous intensity or an equivalent combination

### Primary and secondary outcomes

The primary outcome was all-cause dementia, according to the *Diagnostic and Statistical Manual of Mental Disorders* IV [16]. An independent outcome adjudication committee validated all dementia diagnoses, including a 1-year follow-up period in incident cases to assure there were no false positive diagnoses. Cognition was the secondary outcome measure, which was measured every 2 years with the Mini-Mental State Examination (MMSE) and the Visual Association Test (VAT) [17, 18]. Participants attending at least one follow-up visit were included in the analyses on cognition.

### Statistical analysis

We first assessed the association between the LIBRA index in the preDIVA population and incident dementia with Cox proportional hazards regression. We then divided the study population into participants with a low, intermediate and high LIBRA index based on tertiles of the index [11]. In each group, the crude effect of the intervention on all-cause dementia was assessed with Cox proportional hazards regression (model 1). The years from randomisation to dementia diagnosis or censoring date were used as the timescale. To assess whether the LIBRA index is more useful as a selection tool when containing both modifiable and major non-modifiable risk factors, we repeated our analysis with the LIBRA index expanded with education (model 2) and additionally with age and sex (model 3) [11]. As history of coronary heart disease is not modifiable, we removed it from the LIBRA index in a sensitivity analysis (model 4). Our primary analysis was crude and in a secondary analysis we adjusted for baseline imbalances between the intervention and control groups. We also assessed the effect of adjusting for education, as this is an important risk factor for dementia and is associated with many of the risk/protective factors included in the LIBRA index [3]. The proportional hazards assumption was tested using Schoenfeld residuals and was assessed graphically [19].

Because of the cluster-randomised design we additionally performed a multi-level analysis to account for clustering within general practices and health care centres. To account for competing risk of death, we assessed the intervention effect on mortality in the LIBRA groups, and, when appropriate, performed a competing risk analysis according to the cause-specific hazard method [20]. We added a per-protocol analysis to assess whether the results were influenced by adherence to the intervention or control condition. In the per-protocol analysis, we excluded intervention participants who had on average less than two visits per year and inadvertent crossover control participants who had on average more than two visits per year. As the LIBRA index is more sensitive in a younger cohort [11], we performed a pre-defined subgroup analysis on age (dichotomised at the median). In the primary preDIVA analyses, the intervention seemed to be effective in those individuals with untreated hypertension who adhered to the intervention. However, the LIBRA definition of hypertension is rather crude (dichotomously defined as systolic BP ≥ 140 mmHg, diastolic BP ≥ 90 mmHg and/or use of antihypertensive medication). Therefore, we added subgroup analyses on World Health Organisation hypertension grades (i.e. normotension, systolic BP < 140 mmHg and/or diastolic BP < 90 mmHg; grade I hypertension, systolic BP 140–160 mmHg and/or diastolic BP 90–100 mmHg; grade II or III hypertension, systolic BP ≥ 160 mmHg and/or diastolic BP ≥ 100 mmHg) and use of antihypertensive medication [21]. In the Netherlands, people with a history of cardiovascular disease (CVD) and diabetes visit a practice nurse as part of standard care, potentially diluting an intervention effect [22]. We therefore added analyses in subgroups based on history of CVD and type 2 diabetes. To assess whether the intervention led to an improvement of cardiovascular risk factors, as a proxy for treatment effect, we compared decline in systolic BP, BMI and total cholesterol between baseline and the last available follow-up visit, across the three LIBRA groups.

To assess whether individual changes in cognition vary over time between treatment groups, we used a multilevel growth model stratified for participants with a low, intermediate and high LIBRA index [23]. In this linear mixed-effect model each participant and time in years were considered random effects and a time × randomisation interaction variable was included. Since absolute values of the MMSE and VAT, or logarithmic transformation of these values, were not normally distributed, change in MMSE/VAT since baseline, which was normally distributed, was used as an outcome variable in the model. We performed our analyses in R studio version 3.2 using the survival and nlme packages [24].

## Results

Of the 3526 preDIVA participants at baseline, 3339 (94.7%) had all 10 LIBRA items available at baseline and could be included in the analyses (Additional file 1: Figure S1). The median LIBRA score at baseline was 3.1. Participants with the highest LIBRA index were slightly older (low 74.2 years, intermediate 74.3 years, high 74.5 years; *p* < 0.01) and more often had a low education level (low 19.2%, intermediate 22.1%, high 29.9%; *p* < 0.01; Table 2). Systolic BP was highest in the intermediate LIBRA group (low 151.5 mmHg, intermediate 157.5 mmHg, high 156.7 mmHg; *p* < 0.01). The baseline characteristics of the intervention and control groups within each LIBRA group were well balanced, except for small differences in total cholesterol (respectively, 5.3 vs 5.5 mmol/L; *p* = 0.03) in the intermediate LIBRA group and mean systolic BP (157.9 vs 155.3 mmHg; *p* = 0.04) and sex (37.3% vs 44.2%; *p* = 0.02) in the high LIBRA group (Additional file 1: Table S1).

**Table 2:** Baseline characteristics by LIBRA group

	Low LIBRA index	Intermediate LIBRA index	High LIBRA index	*p* value
Total number of participants	1091	1081	1102	
Range in LIBRA index	−1.0 to 2.6	2.6 to 4.2	4.2 to 11.6	
Demographics				
Age (years)	74.2 (2.5)	74.3 (2.5)	74.5 (2.5)	< 0.01
Sex (male)	528 (48.4%)	519 (48.0%)	445 (40.4%)	< 0.01
Education				< 0.01
Low (< 7 years)	209 (19.2%)	239 (22.1%)	330 (29.9%)	
Medium (7–12 years)	695 (63.7%)	671 (62.1%)	660 (59.9%)	
High (> 12 years)	179 (16.4%)	161 (14.9%)	101 (9.2%)	
Race (white)	1057 (96.9%)	1042 (96.4%)	1054 (95.6%)	< 0.01
Medical history				
CVD (excluding stroke or TIA)	66 (6.0%)	372 (34.4%)	526 (47.7%)	< 0.01
Stroke or TIA	60 (5.5%)	95 (8.8%)	169 (15.3%)	< 0.01
Cardiovascular risk factors				
Systolic BP (mmHg)	151.5 (22.0)	157.5 (20.8)	156.7 (20.6)	< 0.01
Diastolic BP (mmHg)	81.0 (10.9)	82.0 (10.9)	81.3 (11.0)	0.09
Total cholesterol (mmol/L)	5.4 (0.9)	5.4 (1.1)	4.9 (1.2)	< 0.01
LDL cholesterol (mmol/L)	3.3 (0.8)	3.2 (1.0)	2.8 (1.0)	< 0.01
Body mass index (kg/m^2^)	25.9 (3.1)	26.7 (3.6)	29.6 (4.6)	< 0.01
Type 2 diabetes	30 (2.7%)	103 (9.5%)	460 (41.7%)	< 0.01
Smoking (currently)	46 (4.2%)	113 (10.5%)	265 (24.0%)	< 0.01
Alcohol use (units/week)	3 (0–7)	4 (0–14)	0 (0–10)	< 0.01
Physically active (WHO)	1065 (97.6%)	990 (91.6%)	784 (71.1%)	< 0.01
Creatinine (μmol/L)	77 (68–88)	80 (68–93)	82 (71–97)	< 0.01
Medication use				
Antihypertensive medication	332 (30.4%)	631 (58.4%)	838 (76.0%)	< 0.01
Cholesterol-lowering medication	77 (7.1%)	370 (34.2%)	664 (60.3%)	< 0.01
Disability and neuropsychiatric assessment				
Mini-Mental State Examination	29 (28–30)	28,5 (27–29)	28 (27–29)	< 0.01
Visual Association Test	6 (5, 6)	6 (5, 6)	6 (5, 6)	0.05
Geriatric Depression Scale	1 (0–1)	1 (0–2)	2 (0–4)	< 0.01

Data presented as number (percentage), mean (standard deviation) or median (interquartile range)

*LIBRA* LIfestyle for BRAin Health, *CVD* indicates cardiovascular disease *TIA* transient ischemic attack, *BP* blood pressure, *LDL* low-density lipoprotein, *WHO* World Health Organisation

All-cause dementia was diagnosed in 220 (6.7%) participants; 76 of 1091 participants (7.0%) participants with a low LIBRA index, 71 of 1081 (6.6%) with an intermediate LIBRA index and 73 of 1102 participants (6.6%) with a high LIBRA index. The LIBRA index (model 1) was not associated with incident dementia (crude hazard ratio (HR) 1.02 per point increase in LIBRA index, 95% confidence interval (CI) 0.96–1.09). Adding education to the LIBRA index (model 2) did not change these results (HR 1.06; 95% CI 0.90–1.24). The LIBRA index including education, age and sex (model 3) was significantly associated with incident dementia (HR 1.07, 95% CI 1.02–1.12).

**Fig. 1:**
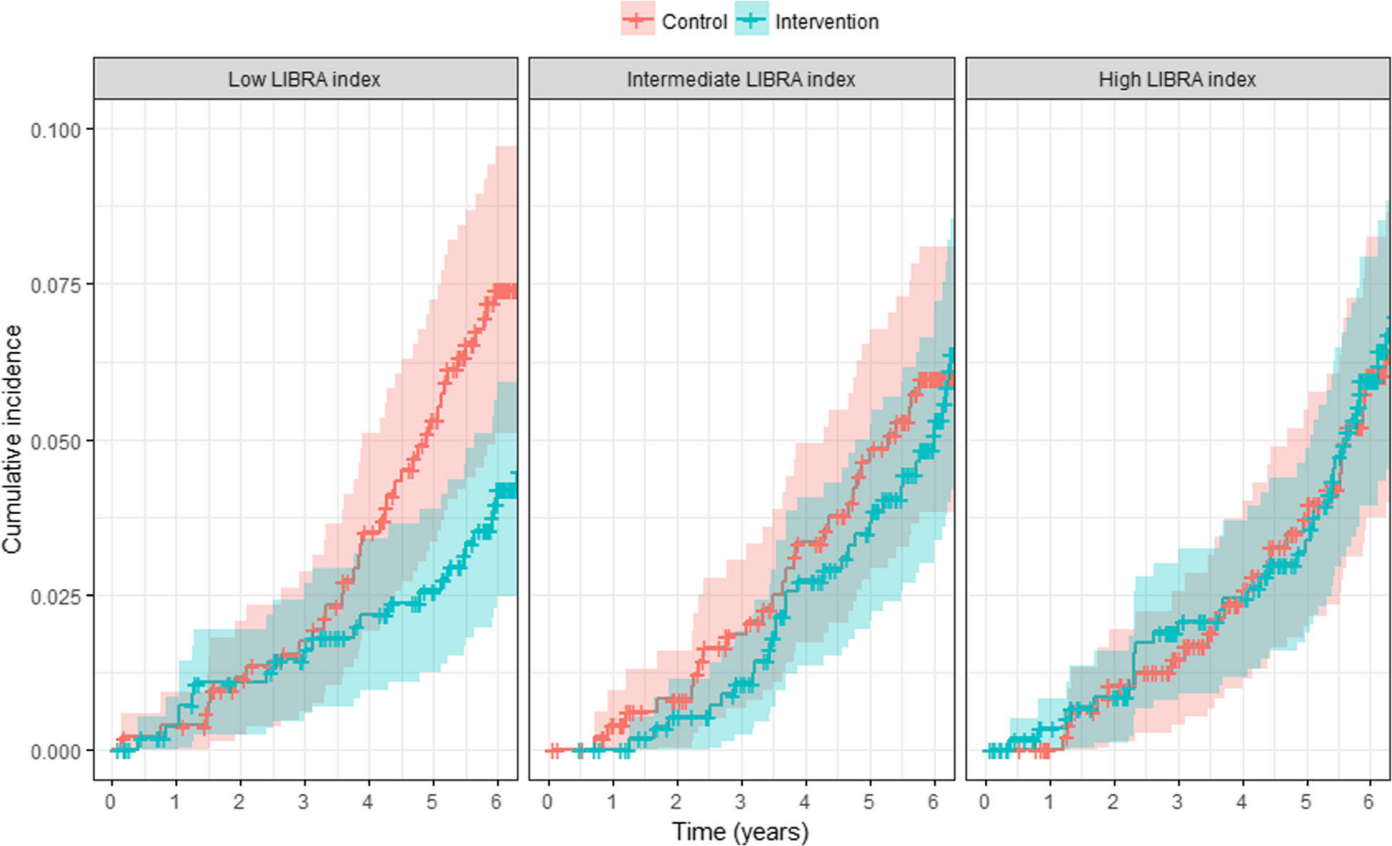
Cumulative incidence curves of risk of dementia comparing intervention and control groups in participants with low, intermediate and high LIBRA index. Line indicates incidence, shaded area indicates 95% CI. Numbers of participants at risk at 6-year follow-up were 791 in low (408 intervention; 383 control), 756 in intermediate (400 intervention; 356 control) and 738 in high (406 intervention, 332 control) LIBRA groups. LIBRA LIfestyle for BRAin Health

The HR of the effect of intensive vascular care on incident all-cause dementia was 0.71 (95% CI 0.45–1.12) in the low, 1.06 (95% CI 0.66–1.69) in the intermediate and 1.02 (95% CI 0.64–1.62) in the high LIBRA groups (model 1; Fig. 1; Table 3). The interaction between randomisation and LIBRA index divided into tertiles was not significant. Also, when including age, sex and education in (models 2 and 3) or excluding coronary heart disease from (model 4) the LIBRA index and stratifying our study population based on this modified LIBRA index, the intervention was not effective in any of the groups (Table 3). Adjustment for baseline imbalances or education did not significantly influence the results, nor did accounting for clustering within general practices and health care centres (Additional file 1: Table S2). The results were similar in the per-protocol analysis (Additional file 1: Table S2). Mortality risk increased with increasing LIBRA index, but the intervention effect on mortality was not significantly different in the LIBRA groups (Additional file 1: Table S3). In all secondary analyses, the HR was lowest, albeit non-significant, in participants with the lowest LIBRA index (Additional file 1: Table S2). Subgroup analyses showed a significant interaction (*p* = 0.03) between age and randomisation in the intermediate LIBRA group with a lower HR in younger participants aged < 74.3 years (HR 0.55; 95% 0.26–1.17) compared to older participants (HR 1.65; 95% CI 0.88–3.09) (Additional file 1: Table S4). We found an interaction with diabetes in the high LIBRA group (*p* = 0.03), with a lower HR in participants with diabetes (HR 0.61; 95% CI 0.32–1.15) in comparison to those without (HR 1.78; 95% CI 0.87–3.64). We found no other interactions in the subgroup analyses. Participants with a higher LIBRA index had on average more decline in systolic BP (respectively in the low, intermediate and high LIBRA groups − 2.3, − 5.9 and − 5.8; *p* < 0.01), less decline in total cholesterol (− 0.3, − 0.3 and − 0.1; *p* < 0.01) and more decline in BMI (− 0.5, − 0.5 and − 0.9; *p* < 0.01). The intervention led to a significant decline in systolic BP in the low (intervention vs control − 3.9 vs − 0.5; *p* = 0.03) and intermediate (− 7.4 vs − 4.2; *p* = 0.04) LIBRA groups, but not in the high LIBRA group (− 7.1 vs − 4.3; *p* = 0.09; Additional file 1: Table S5). The intervention did not significantly reduce cholesterol or BMI in any of the LIBRA groups (Additional file 1: Table S5).

**Table 3:** Intervention effect on incident all-cause dementia across the models, by LIBRA group

	LIBRA group	Intervention, *n*/*N* (%)	Control, *n*/*N* (%)	Hazard ratio (95% CI)	*p* value for interaction
Model 1: LIBRA index	Low	33/567 (5.8%)	43/524 (8.2%)	0.71 (0.45–1.12)	Ref.
	Intermediate	39/576 (6.8%)	32/505 (6.3%)	1.06 (0.66–1.69)	0.23
	High	41/606 (6.8%)	32/496 (6.5%)	1.02 (0.64–1.62)	0.27
Model 2: LIBRA index including education	Low	28/555 (5.0%)	39/498 (7.8%)	0.64 (0.40–1.05)	Ref.
	Intermediate	38/525 (7.2%)	31/482 (6.4%)	1.11 (0.69–1.79)	0.12
	High	46/660 (7.0%)	35/525 (6.7%)	1.03 (0.66–1.59)	0.17
Model 3: LIBRA index including age, sex and education	Low	32/564 (5.7%)	33/515 (6.4%)	0.88 (0.54–1.43)	Ref.
	Intermediate	35/568 (6.2%)	34/510 (6.7%)	0.91 (0.57–1.47)	0.94
	High	45/608 (7.4%)	38/480 (7.9%)	0.92 (0.59–1.41)	0.92
Model 4: LIBRA index excluding coronary heart disease	Low	36/559 (6.0%)	45/559 (8.1%)	0.75 (0.48–1.16)	Ref.
	Intermediate	35/570 (6.1%)	28/477 (5.9%)	1.05 (0.64–1.72)	0.32
	High	42/580 (7.2%)	34/489 (7.0%)	1.01 (0.64–1.59)	0.34

*LIBRA* LIfestyle for BRAin Health, *CI* confidence interval, *Ref*. reference category

**Fig. 2:**
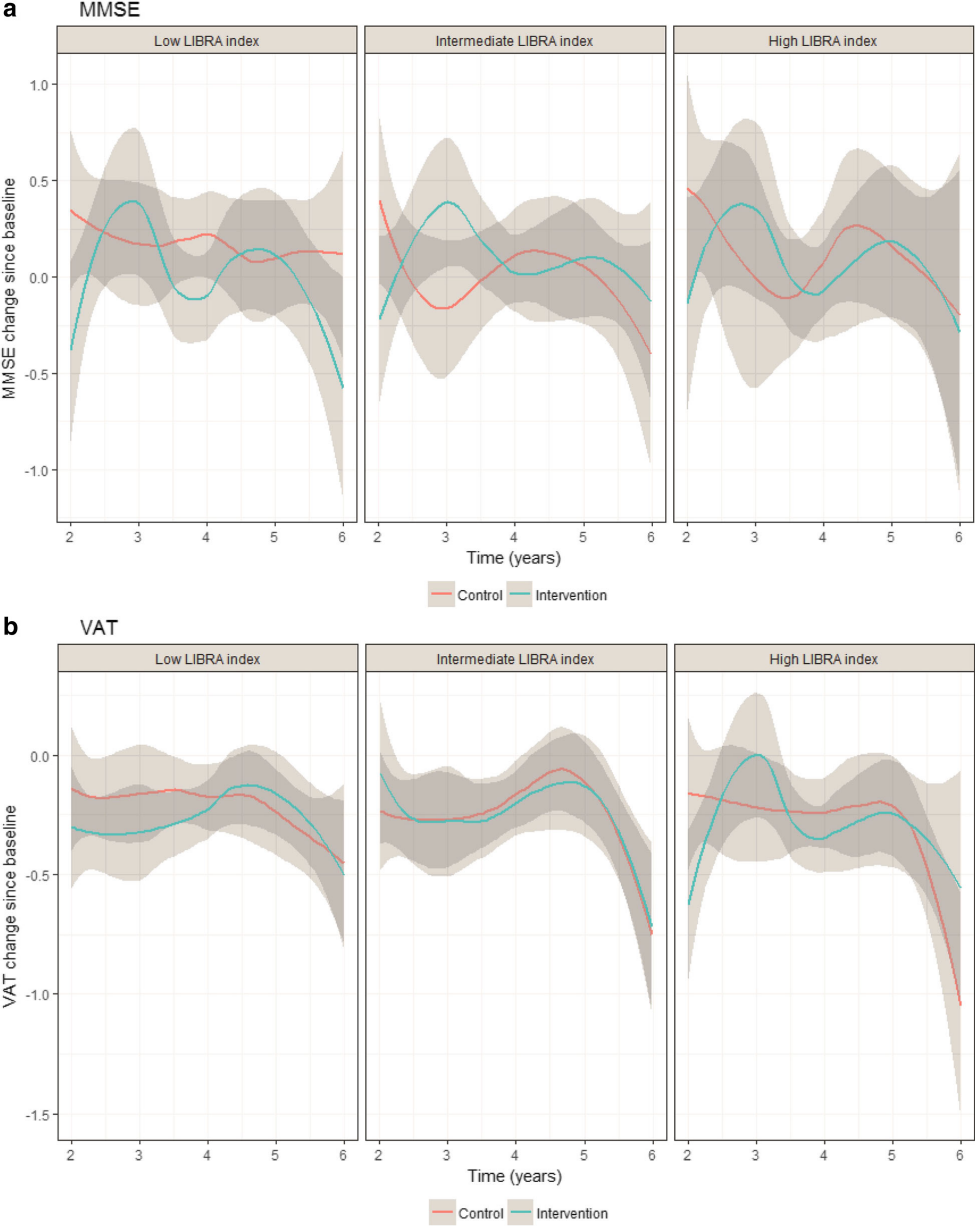
Effect of intervention on MMSE (**a**) and VAT (**b**) change since baseline in LIBRA groups. Trajectories of change in MMSE and VAT since baseline comparing control group (red line) to intervention group (blue line) in each LIBRA group, as predicted with multilevel growth model. Positive value indicates increase in MMSE/VAT since baseline, negative value indicates decrease. LIBRA LIfestyle for BRAin Health, MMSE Mini-Mental State Examination, VAT Visual Association Test

A total of 2674 participants had at least one valid MMSE score and 2671 participants at least one valid VAT score after baseline and could be included in the analyses on cognitive decline (Additional file 1: Figure S1). Participants excluded from these analyses were on average older, had a higher cardiovascular risk and had a lower baseline MMSE and VAT (Additional file 1: Table S6). After 3 years, decline in MMSE did not significantly differ between the intervention and control groups among participants with a low (mean difference (MD) − 0.08; 95% CI − 0.28 to 0.13), intermediate (MD 0.07; 95% CI − 0.14 to 0.27) or high (MD − 0.06; 95% CI − 0.30 to 0.18; Fig. 2a, Additional file 1: Table S7) LIBRA index. Decline in VAT also did not differ between treatment groups in the low (MD 0.03; 95% CI − 0.09 to 0.14), intermediate (MD − 0.04; 95% CI − 0.16 to 0.08) or high (MD 0.07; 95% CI − 0.05 to 0.19; Fig. 2b, Additional file 1: Table S7) LIBRA groups.

## Discussion

In the preDIVA study population, aged 70–78 years, the LIBRA index did not identify a high-risk group in whom the multi-domain intervention was effective in preventing dementia or cognitive decline. On the contrary, there was a trend for a preventive effect in the subgroup with a low LIBRA index. Results were comparable when including non-modifiable risk factors in the LIBRA index.

The concept of selecting people at increased risk of dementia for preventive interventions to magnify the intervention effect is widely supported among experts in the field and has been incorporated in the design of recent multi-domain prevention trials [6, 7]. Our results do not support this strategy, and are even in contrast with this concept, at least in later life, suggesting a more favourable effect of the intervention in those with a low LIBRA index. A potential explanation for this is that the contrast between the intervention and control conditions was too small, partly due to Hawthorne effects and improvements in the standard care for cardiovascular risk management during the trial [5]. Although a greater reduction in systolic BP could be achieved in participants with a higher LIBRA index, this was the case in both the intervention and control groups and the difference between the treatment groups was smallest in the high LIBRA group. Another potential explanation for our results is that the LIBRA index does not successfully classify dementia risk in this older population aged 70–78 years. Indeed, our analyses did not show an association between a high LIBRA index and increased risk of dementia. Since preDIVA is an RCT, this could potentially (partly) be due to the fact that the dementia risk was influenced during the trial by the intervention and/or Hawthorne effects [5]. For example, systolic BP decreased by approximately 8 mmHg in the intervention group and 4 mmHg in the control group, and the decline was steepest in participants with hypertension at baseline [5]. In one of the LIBRA validation studies, a higher LIBRA index was associated (at group level) with an increased risk of dementia in people aged 70–79 years [11]. The individual predictive accuracy in late life was poor, however, with a *C* statistic of 0.50, and seemed to decrease with increasing age. Investigating the utility of the LIBRA index as a selection tool for prevention trials at a younger age (55–70 years) may yield different results. A third potential explanation is that the factors in the LIBRA index and in other dementia risk scores are dichotomous and not designed to precisely quantify the magnitude of the risk/protective factor or the room for improvement. For example, the potential for improvement is different for someone with a systolic BP of 125 mmHg on antihypertensive medication compared to a person with a systolic BP of 155 mmHg without medication, although both are weighted equally in the LIBRA index with the dichotomous score for hypertension (including both high BP and/or antihypertensive medication use). In order for a risk estimation tool to be useful for selection of high-risk populations for dementia prevention trials, the potential for improvement should be taken into account (e.g. by distinguishing treated or untreated hypertension).

Regardless of the LIBRA index performance in high-age populations, the concept of selecting people at high risk of dementia may only be appropriate for younger people (i.e. age < 70 years). In older people at high risk of dementia, cerebrovascular and neurodegenerative damage may already be irreversible, while those with a low risk could still benefit from risk factor improvement in order to maintain cognitive function. Also, several observational studies have shown a diminishing or even inverting association between risk factors and incident dementia in older people, as for example the J-shaped relation with BP [25]. Therefore, future trials should perhaps either focus on people with lowest dementia risk in old age or highest dementia risk in midlife. This would, however, imply that substantially larger sample sizes or longer follow-up will be required, as incidence rates in these groups are lower.

A strength of this analysis is that preDIVA is, up until now, the only multi-domain prevention trial with dementia as the primary outcome. The population-based approach with few exclusion criteria renders preDIVA a suitable study to test whether the LIBRA index is a promising tool to select high-risk groups from the general population. A limitation is the overall neutral result of the preDIVA trial, perhaps limiting the possibility to detect high-risk groups who benefit most. However, a significant effect of the intervention was found in the per-protocol analysis among participants with untreated hypertension at baseline (HR 0.54, 95% CI 0.32–0.92) [5], while the results of the present analyses do not show a trend towards improved treatment effects in higher LIBRA groups. Another limitation is that no other neuropsychological tests were performed besides the MMSE and VAT to detect more subtle cognitive changes. We did not have information on two of the 12 LIBRA items, including cognitive activity which is the strongest-weighted item in the LIBRA index [9]. These factors were, however, already identified as risk factors that need further validation in the systematic review and Delphi consensus used to design the LIBRA index, and were also not included in the validation study among people in late life (70–79 years) [11, 12]. Furthermore, it may be argued that cognitive activity at this age is not as much a modifiable risk factor but rather an early indicator of developing cognitive decline and dementia [26].

## Conclusions

Within our study population of community-dwelling people aged 70–78 years, a modifiable dementia risk score does not identify heterogeneity in the treatment effect of a multi-domain intervention to prevent dementia or cognitive decline. This suggests that in older adults a high LIBRA index may not be a suitable parameter to select participants for a dementia prevention trial. Specific characteristics of the preDIVA study, including the overall neutral effect of the intervention and relatively high age group, may have contributed to the lack of discriminating capacity of the LIBRA index.

## Additional file

Additional file 1: **Figure S1.** Flow diagram. **Table S1.** Baseline characteristics per randomisation group and LIBRA group. **Table S2.** Secondary analyses. **Table S3.** Competing risk analysis. **Table S4.** Subgroup analyses on age, hypertension grade, antihypertensive medication, history of cardiovascular disease and diabetes. **Table S5.** Treatment effect on vascular risk factors in LIBRA groups. **Table S6.** Baseline characteristics of participants included in and excluded from cognitive analyses. **Table S7.** Treatment effect on cognition in LIBRA groups (DOCX 533 kb)

## Abbreviations

BMI: Body mass index
BP: Blood pressure
CI: Confidence interval
CVD: Cardiovascular disease
HR: Hazard ratio
ISRCTN: International Standard Randomised Controlled Trial Number
LIBRA: LIfestyle for BRAin Health
MD: Mean difference
MMSE: Mini-Mental State Examination
preDIVA: Prevention of Dementia by Intensive Vascular Care
RCT: Randomised controlled trial
VAT: Visual Association Test
