# Supplementary material for: Modifiable dementia risk score to study heterogeneity in treatment effect of a dementia prevention trial: a post hoc analysis in the preDIVA trial using the LIBRA index
Source: Alzheimers Res Ther. 2018 Jun 30;10:62. doi: 10.1186/s13195-018-0389-4 (PMC6026510; doi:10.1186/s13195-018-0389-4)
Supplement: Supplementary file 1 — Figure S1. Flow diagram. Table S1. Baseline characteristics per randomisation group and LIBRA group. Table S2. Secondary analyses. Table S3. Competing risk analysis. Table S4. Subgroup analyses on age, hypertension grade, antihypertensive medication, history of cardiovascular disease and diabetes. Table S5. Treatment effect on vascular risk factors in LIBRA groups. Table S6. Baseline characteristics of participants included in and excluded from cognitive analyses. Table S7. Treatment effect on cognition in LIBRA groups (DOCX 533 kb) [file 13195_2018_389_MOESM1_ESM.docx]

**Additional file 1**

**Supplement to**

*“Modifiable dementia risk score to study heterogeneity in treatment effect of a dementia prevention trial: a post hoc analysis in the preDIVA trial using the LIBRA index”*

*T. van Middelaar, M.P. Hoevenaar-Blom, W.A. van Gool, E.P. Moll van Charante, J. van Dalen, K. Deckers, S. Köhler, E. Richard*

**Table of contents**

|  |  | Page |
| --- | --- | --- |
| Figure S1 | Flow diagram | 2 |
| Table S1 | Baseline characteristics per randomisation group and LIBRA group | 3 |
| Table S2 | Secondary analyses | 5 |
| Table S3 | Competing risk analysis | 7 |
| Table S4 | Subgroup analyses on age, hypertension grade, antihypertensive medication, history of cardiovascular disease and diabetes | 8 |
| Table S5 | Treatment effect on vascular risk factors in the LIBRA groups | 9 |
| Table S6 | Baseline characteristics of participant in- and excluded in the cognitive analyses | 11 |
| Table S7 | Treatment effect on cognition in the LIBRA groups | 13 |

**Figure S1. Flow diagram**


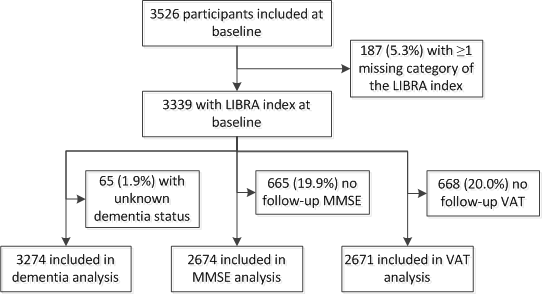


*LIBRA indicates lifestyle for brain health; MMSE, mini-mental state examination; VAT, visual association test.*

**Table S1. Baseline characteristics per randomisation group and LIBRA risk group**

|  |  | | Low LIBRA index (N=1091) | | | | | Intermediate LIBRA index (N=1081) | | | | | High LIBRA index (N=1102) | | | | | |
| --- | --- | --- | --- | --- | --- | --- | --- | --- | --- | --- | --- | --- | --- | --- | --- | --- | --- | --- |
|  |  | | **Control** | | **Intervention** | | **P** | **Control** | | **Intervention** | | **P** | **Control** | | **Intervention** | | | **P** |
| Demographics | | |  |  |  |  |  |  |  |  |  |  |  |  |  |  | |  |
|  | | Age (years) | 74.2 | (SD 2.5) | 74.1 | (SD 2.5) | 0.50 | 74.4 | (SD 2.5) | 74.3 | (SD 2.4) | 0.52 | 74.4 | (SD 2.51 | 74.5 | (SD 2.43 | | 0.58 |
|  | | Gender (male) | 258 | (49.2%) | 270 | (47.6%) | 0.62 | 236 | (46.7%) | 283 | (49.1%) | 0.46 | 219 | (44.2%) | 226 | (37.3%) | | 0.02 |
|  | | Education |  |  |  |  | 0.59 |  |  |  |  | 1.00 |  |  |  |  | | 0.36 |
|  | | Low (<7 yr) | 95 | (18.1%) | 114 | (20.1%) |  | 112 | (22.2%) | 127 | (22.0%) |  | 145 | (29.2%) | 185 | (30.5%) | |  |
|  | | Medium (7-12 yr) | 340 | (64.9%) | 355 | (62.6%) |  | 314 | (62.2%) | 357 | (62.0%) |  | 290 | (58.5%) | 370 | (61.1%) | |  |
|  | | High (>12 yr) | 82 | (15.6%) | 97 | (17.1%) |  | 75 | (14.9%) | 86 | (14.9%) |  | 52 | (10.5%) | 49 | (8.1%) | |  |
|  | | Race (white) | 550 | (97.0%) | 507 | (96.8%) | 1.00 | 549 | (95.3% | 493 | (97.6%) | 0.24 | 582 | (96.0%) | 472 | (95.2%) | | 0.59 |
| Medical history | | |  |  |  |  |  |  |  |  |  |  |  |  |  |  | |  |
|  | | CVD (excl. stroke or TIA) | 36 | (6.9%) | 30 | (5.3%) | 0.31 | 167 | (33.1%) | 205 | (35.6%) | 0.40 | 240 | (48.4%) | 286 | (47.2%) | | 0.72 |
|  | | Stroke or TIA | 30 | (5.7%) | 30 | (5.3%) | 0.79 | 45 | (8.9%) | 50 | (8.7%) | 0.91 | 85 | (17.1%) | 84 | (13.9%) | | 0.15 |
| Cardiovascular risk factors | | |  |  |  |  |  |  |  |  |  |  |  |  |  |  | |  |
|  | SBP (mmHg) | | 150.8 | (SD 20.6) | 152.2 | (SD 23.3) | 0.29 | 156.6 | (SD 20.7) | 158.3 | (SD 20.9) | 0.18 | 155.3 | (SD 19.7) | 157.9 | | (SD 21.2) | 0.04 |
|  | DBP (mmHg) | | 81.0 | (SD 10.2) | 81.0 | (SD 11.4) | 1.00 | 81.9 | (SD 10.9) | 82.0 | (SD 10.8) | 0.84 | 81.3 | (SD 10.9) | 81.3 | | (SD 11.1) | 0.92 |
|  | Total cholesterol (mmol/L) | | 5.4 | (SD 0.9) | 5.4 | (SD 0.9) | 0.48 | 5.5 | (SD 1.1) | 5.3 | (SD 1.1) | 0.03 | 4.9 | (SD 1.2) | 4.9 | | (SD 1.2) | 0.62 |
|  | LDL cholesterol (mmol/L) | | 3.2 | (SD 0.8) | 3.3 | (SD 0.8) | 0.42 | 3.1 | (SD 1.0) | 3.3 | (SD 1.0) | 0.05 | 2.7 | (SD 1.0) | 2.8 | | (SD 1.1) | 0.12 |
|  | BMI (kg/m^2^) | | 25.9 | (SD 3.1) | 26.0 | (SD 3.1) | 0.47 | 26.7 | (SD 3.6) | 26.8 | (SD 3.7) | 0.71 | 29.5 | (SD 4.61) | 29.7 | | (SD 4.6) | 0.56 |
|  | Type 2 diabetes | | 12 | (2.3%) | 18 | (3.2%) | 0.47 | 47 | (9.3%) | 56 | (9.7%) | 0.83 | 206 | (41.5%) | 254 | | (41.9%) | 0.91 |
|  | Smoking (currently) | | 20 | (3.8%) | 26 | (4.6%) | 0.52 | 53 | (10.5%) | 60 | (10.4%) | 1.00 | 120 | (24.2%) | 145 | | (23.9%) | 0.94 |
|  | Alcohol use (units/week) | | 3 | [0-8] | 3 | [0-7] | 0.87 | 4 | [0-14] | 4 | [0-14] | 0.70 | 1 | [0-10] | 0 | | [0-12] | 0.55 |
|  | Physically active (WHO) | | 550 | (97.0%) | 515 | (98.3%) | 0.21 | 531 | (92.2%) | 459 | (90.9%) | 0.52 | 424 | (70%) | 360 | | (72.6%) | 0.36 |
|  | Creatinine (umol/L) | | 76 | [68-88] | 77 | [68-88] | 0.98 | 79 | [67-93] | 80 | [69-93] | 0.35 | 81 | [70-97] | 82 | | [72-97] | 0.41 |
| Medication use | | |  |  |  |  |  |  |  |  |  |  |  |  |  |  | |  |
|  | Antihypertensive med. | | 161 | (30.7%) | 171 | (30.2%) | 0.84 | 310 | (61.4%) | 321 | (55.7%) | 0.08 | 384 | (77.4%) | 454 | | (74.9%) | 0.38 |
|  | Cholesterol lowering med. | | 38 | (7.3%) | 39 | (6.9%) | 0.82 | 171 | (33.9%) | 199 | (34.5%) | 0.81 | 299 | (60.3%) | 365 | | (60.2%) | 1.00 |
| Disability and neuropsychiatric assessment | | | | | | | | | | | | | | | | | | |
|  | MMSE | | 29 | [28-29] | 29 | [27-30] | 0.58 | 29 | [27-29] | 28 | [27-29] | 0.99 | 28 | [27-29] | 28 | | [27-29] | 0.44 |
|  | VAT | | 6 | [5-6] | 6 | [5-6] | 0.60 | 6 | [5-6] | 6 | [5-6] | 0.62 | 6 | [5-6] | 6 | | [5-6] | 0.20 |
|  | GDS | | 1 | [0-1] | 1 | [0-2] | 0.44 | 1 | [0-2] | 1 | [0-2] | 0.67 | 2 | [0-4] | 2 | | [0-4] | 0.27 |

*Data are presented as number (percentage), mean (standard deviation) or median [interquartile range]. Yr indicates years; CVD, cardiovascular disease; excl., excluding; TIA, transient ischemic attack; SBP, systolic blood pressure; DBP, diastolic blood pressure; LDL, low-density lipoprotein; BMI, body-mass index; WHO, world health organisation; med., medication; MMSE, mini-mental state examination; VAT, visual association test; GDS, geriatric depression scale.*

**Table S2. Secondary analyses**

|  |  | **Intervention (n,%)** | **Control (n,%)** | **Hazard ratio (95% CI)** | **p-for interaction** |
| --- | --- | --- | --- | --- | --- |
| **Adjusting for total cholesterol** | Low | 33/567 (5.8%) | 43/524 (8.2%) | 0.71 (0.45-1.12) | Ref |
|  | Intermediate | 39/576 (6.8%) | 32/505 (6.3%) | 1.05 (0.66-1.68) | 0.24 |
|  | High | 41/606 (6.8%) | 32/496 (6.5%) | 1.01 (0.64-1.60) | 0.27 |
| **Adjusting for mean systolic BP** | Low | 33/567 (5.8%) | 43/524 (8.2%) | 0.72 (0.46-1.13) | Ref |
|  | Intermediate | 39/576 (6.8%) | 32/505 (6.3%) | 1.06 (0.66-1.69) | 024 |
|  | High | 41/606 (6.8%) | 32/496 (6.5%) | 1.04 (0.65-1.65) | 0.26 |
| **Adjusting for gender** | Low | 33/567 (5.8%) | 43/524 (8.2%) | 0.71 (0.45-1.12) | Ref |
|  | Intermediate | 39/576 (6.8%) | 32/505 (6.3%) | 1.06 (0.66-1.69) | 0.24 |
|  | High | 41/606 (6.8%) | 32/496 (6.5%) | 1.01 (0.63-1.60) | 0.27 |
| **Adjusting for education** | Low | 33/567 (5.8%) | 43/524 (8.2%) | 0.70 (0.44-1.10) | Ref |
|  | Intermediate | 39/576 (6.8%) | 32/505 (6.3%) | 1.03 (0.64-1.65) | 0.25 |
|  | High | 41/606 (6.8%) | 32/496 (6.5%) | 105 (0.66-1.69) | 0.21 |
| **Accounting for clustering*** | Low | 33/567 (5.8%) | 43/524 (8.2%) | 0.71 (0.45-1.13) | Ref |
|  | Intermediate | 39/576 (6.8%) | 32/505 (6.3%) | 1.06 (0.66-1.69) | 0.22 |
|  | High | 41/606 (6.8%) | 32/496 (6.5%) | 1.05 (0.64-1.72) | 0.26 |
| **Per-protocol analysis** | Low | 24/429 (5.6%) | 41/480 (8.5%) | 0.62 (0.37-1.02) | Ref |
|  | Intermediate | 24/452 (5.3%) | 32/466 (6.9%) | 0.71 (0.42-1.21) | 0.73 |
|  | High | 30/443 (6.8) | 29/463 (6.3%) | 1.02 (0.61-1.71) | 0.17 |

** Clusters were* general practices and health-care centres. *The p-for interaction indicates the p-value of the interaction variable intervention*LIBRA group. The p-for interaction for the intermediate LIBRA group compares the low with the intermediate LIBRA group; the p-for interaction for the high LIBRA group compares the low with the high LIBRA group. CI indicates confidence interval; Ref, reference category; BP, blood pressure.*

**Table S3. Competing risk analysis**

|  | **Dementia-free survival (CSHR, 95% CI)** | **Mortality (CSHR, 95% CI)** | **Dementia (CSHR, 95% CI)** | **Dementia (SHR, 95% CI)** |
| --- | --- | --- | --- | --- |
| **Low risk** | 1.04 (0.91-1.19) | 1.07 (0.74-1.56) | 0.71 (0.45-1.12) | 0.70 (0.44-1.10) |
| **Intermediate risk** | 0.94 (0.83-1.08) | 1.12 (0.81-1.54) | 1.06 (0.66-1.69) | 1.06 (0.67-1.70) |
| **High risk** | 0.95 (0.83-1.09) | 0.87 (0.68-1.12) | 1.03 (0.65-1.63) | 1.05 (0.66-1.67) |

*CSHR indicates cause specific hazard ratio, an estimate for the direct effect of the intervention on survival, mortality or dementia. SHR indicates subdistribution hazard ratio, an estimate for the risk of dementia while accounting for mortality as competing event. This is done by giving every participant with no diagnosis of dementia the longest follow-up duration instead censoring them at time of death or lost to follow-up.*

**Table S4. Subgroup analyses on age, hypertension grade, antihypertensive medication, history of cardiovascular disease and diabetes**

|  | **Low risk** | | | **Intermediate risk** | | | **High risk** | | |
| --- | --- | --- | --- | --- | --- | --- | --- | --- | --- |
|  | **N=** | **HR (95% CI)** | **p-for int.** | **N=** | **HR (95% CI)** | **p-for int.** | **N=** | **HR (95% CI)** | **p-for int.** |
| **Age <74.3 year** | 584 | 0.96 (0.44-2.10) | Ref | 554 | 0.55 (0.26-1.17) | Ref | 541 | 1.13 (0.52-2.47) | Ref |
| **Age ≥74.3 year** | 526 | 0.63 (0.36-1.11) | 0.40 | 546 | 1.65 (0.88-3.09) | 0.03 | 588 | 0.95 (0.54-1.69) | 0.73 |
| **Normotension** | 366 | 0.90 (0.44-1.82) | Ref | 184 | 1.07 (0.24-4.77) | Ref | 209 | 0.92 (0.34-2.45) | Ref |
| **Grade I hypertension** | 364 | 0.53 (0.20-1.40) | 0.37 | 451 | 1.00 (0.50-2.01) | 0.72 | 454 | 1.01 (0.48-2.09) | 0.87 |
| **Grade II or III hypertension** | 380 | 0.59 (0.27-1.28) | 0.43 | 465 | 1.05 (0.52-2.13) | 0.74 | 466 | 1.18 (0.55-2.51) | 0.71 |
| **AHM** | 337 | 1.01 (0.48-2.16) | Ref | 642 | 1.12 (0.62-2.04) | Ref | 858 | 1.01 (0.59-1.70) | Ref |
| **No AHM** | 770 | 0.58 (0.33-1.04) | 0.27 | 457 | 0.91 (0.42-1.98) | 0.64 | 270 | 1.06 (0.40-2.78) | 0.93 |
| **History of CVD** | 123 | 0.87 (0.24-3.16) | Ref | 661 | 1.25 (0.62-2.53) | Ref | 508 | 0.82 (0.44-1.53) | Ref |
| **No history of CVD** | 981 | 0.68 (0.42-1.12) | 0.79 | 431 | 0.91 (0.47-1.75) | 0.53 | 614 | 1.32 (0.65-2.69) | 0.32 |
| **Diabetes** | 30 | NA |  | 104 | 0.77 (0.22-2.69) | Ref | 470 | 0.61 (0.32-1.15) | Ref |
| **No diabetes** | 1080 | NA |  | 996 | 1.12 (0.68-1.87) | 0.49 | 659 | 1.78 (0.87-3.64) | 0.03 |

*History of cardiovascular disease is defined as a previous diagnosis of myocardial infarction, angina, stroke, TIA and/or peripheral arterial disease. P for int. indicates the p-value for the interaction variable intervention*subgroup. Diabetes subgroup analysis could not be performed in the low LIBRA group, as numbers were too small. Ref indicates the reference category; AHM, antihypertensive medication; NA, not applicable.*

**Table S5. Treatment effect on vascular risk factors in the LIBRA groups**

|  | Low risk | | | Intermediate risk | | | High risk | | |
| --- | --- | --- | --- | --- | --- | --- | --- | --- | --- |
|  | **Intervention** | **Control** | **p-value** | **Intervention** | **Control** | **p-value** | **Intervention** | **Control** | **p-value** |
| SBP (mmHg) | -3.9 (23.4) | -0.5 (21.9) | 0.03 | -7.4 (24.2) | -4.2 (21.7) | 0.04 | -7.1 (25.5) | -4.3 (22.8) | 0.09 |
| Cholesterol (mmol/L) | -0.4 (0.9) | -0.3 (0.9) | 0.11 | -0.3 (1.1) | -0.4 (1.0) | 0.07 | -0.12 (1.0) | -0.17 (1.1) | 0.54 |
| BMI (kg/m^2^) | -0.5 (4.2) | -0.5 (2.8) | 0.82 | -0.5 (2.7) | -0.6 (2.8) | 0.47 | -1.0 (3.9) | -0.8 (3.7) | 0.64 |

*Data presented are mean (SD) change in systolic BP, BMI or cholesterol between baseline and the last available follow-up visit, comparing the intervention to the control group. BP indicates blood pressure; BMI, body mass index.*

**Table S6. Baseline characteristics of participant in- and excluded in the cognitive analyses**

|  |  | **Included in analyses**  **(N= 2674)** | | **Excluded from analyses**  **(N= 665)** | | **P-value** |
| --- | --- | --- | --- | --- | --- | --- |
| **Demographics** | |  |  |  |  |  |
|  | Age (years) | 74.2 | (SD 2.4) | 74.8 | (SD 2.5) | <0.01 |
|  | Sex (male) | 1214 | (45.4%) | 310 | (46.6%) | 0.60 |
|  | Education |  |  |  |  | <0.01 |
|  | Low (<7 years) | 596 | (22.3%) | 195 | (29.3%) |  |
|  | Medium (7-12 years) | 1690 | (63.2%) | 375 | (56.4%) |  |
|  | High (>12 years) | 367 | (13.7%) | 86 | (12.9%) |  |
|  | Race (white) | 2577 | (96.4%) | 641 | (96.4%) | 0.35 |
| **Medical history** | |  |  |  |  |  |
|  | CVD (excl. stroke or TIA) | 763 | (28.5%) | 228 | (34.3%) | <0.01 |
|  | Stroke or TIA | 257 | (9.6%) | 76 | (11.4%) | 0.15 |
| **Cardiovascular risk factors** | | |  |  |  |  |
|  | Systolic BP (mmHg) | 154.7 | (SD 21.0) | 157.7 | (SD 22.7) | <0.01 |
|  | Diastolic BP (mmHg) | 81.3 | (SD 10.7) | 82.0 | (SD 11.8) | 0.21 |
|  | Total cholesterol (mmol/L) | 5.3 | (SD 1.1) | 5.2 | (SD 1.1) | 0.03 |
|  | LDL cholesterol (mmol/L) | 3.1 | (SD 1.0) | 3.0 | (SD 0.9) | 0.04 |
|  | Body mass index (kg/m^2^) | 27.4 | (SD 4.2) | 27.4 | (SD 4.2) | 0.96 |
|  | Type 2 diabetes | 502 | (18.8%) | 102 | (15.3%) | 0.04 |
|  | Smoking (currently) | 334 | (12.5%) | 105 | (15.8%) | 0.02 |
|  | Alcohol use (units/week) | 3 | [0-10] | 2 | [0-10] | 0.09 |
|  | Physically active (WHO) | 2353 | (88%) | 543 | (81.7%) | <0.01 |
|  | Creatinine (umol/L) | 79 | [69-92] | 81 | [70-94] | 0,0308 |
| **Medication use** | |  |  |  |  |  |
|  | Antihypertensive medication | 1466 | (54.8%) | 371 | (55.8%) | 0.65 |
|  | Cholesterol lowering medication | 905 | (33.8%) | 228 | (34.3%) | 0.85 |
| **Disability and neuropsychiatric assessment** | | | | |  |  |
|  | Mini-mental State Examination (MMSE) | 29 | [27-29] | 28 | [27-29] | <0.01 |
|  | Visual Association Test (VAT) | 6 | [5-6] | 6 | [5-6] | <0.01 |
|  | Geriatric Depression Scale (GDS) | 1 | [0-2] | 1 | [0-3] | <0.01 |

*Participants are excluded from the analyses on cognitive decline if they only had one MMSE/VAT. Data are presented as number (percentage), mean (standard deviation) or median [interquartile range]. CVD indicates cardiovascular disease; excl., excluding; TIA, transient ischemic attack; BP, blood pressure; LDL, low-density lipoprotein; WHO, world health organisation.*

**Table S7. Treatment effect on cognitive decline since baseline in the LIBRA risk groups**

|  |  | Low LIBRA index  (beta, 95% CI) | Intermediate LIBRA index  (beta, 95% CI) | High LIBRA index  (beta, 95% CI) |
| --- | --- | --- | --- | --- |
| MMSE decline | **Intercept (at 3 years follow-up)** | 0.09 (-0.06 to 0.24) | 0.00 (-0.15 to 0.14) | 0.00 (-0.18 to 0.17) |
|  | **Randomisation** | -0.08 (-0.28 to 0.13) | 0.07 (-0.14 to 0.27) | -0.06 (-0.30 to 0.18) |
|  | **Time (years)** | -0.06 (-0.10 to -0.01) | -0.04 (-0.09 to 0.01) | -0.07 (-0.13 to -0.02) |
|  | **Randomisation * Time** | 0.01 (-0.05 to 0.07) | 0.01 (-0.06 to 0.07) | 0.03 (-0.05 to 0.10) |
| VAT decline | **Intercept (at 3 years follow-up)** | -0.22 (-0.30 to -0.14) | -0.28 (-0.37 to -0.19) | -0.25 (-0.34 to -0.16) |
|  | **Randomisation** | -0.03 (-0.14 to 0.09) | 0.04 (-0.08 to 0.16) | -0.07 (-0.19 to 0.05) |
|  | **Time (years)** | -0.09 (-0.14 to -0.05) | -0.01 (-0.05 to 0.04) | -0.10 (-0.16 to -0.04) |
|  | **Randomisation * Time** | 0.05 (-0.01 to 0.12) | -0.08 (-0.15 to -0.01) | 0.02 (-0.06 to 0.10) |

*The intercept indicates the mean change in MMSE/VAT since baseline at 3 years follow-up for the control group. Randomisation indicates the mean difference in MMSE/VAT for the intervention compared to the control group at year 3 years. Time (years) indicates mean change in MMSE/VAT in the control group per year. The interaction term (Randomisation * Time) describes the difference in the effect of time for the intervention compared to the control group.*
